# A Case of Intussusception with Occlusion

**Published:** 1886-08-20

**Authors:** J. P. Motley

**Affiliations:** Daviston, Ala.


### A CASE OF INTUSSUSCEPTION WITH OCCLUSION.

BY J. P. MOTLEY, M. D., OF DAVISTON, ALA.

Mrs. C—— was attacked with severe retching and vomiting, which lasted for about twelve hours despite every effort to prevent it. Seemingly, the patient was much exhausted ; had complained for several days of a feeling, as she expressed it, that her bowels were slipping into one another. At my first visit—April 1—I detected what I at first supposed to be a fæcal mass ; but upon further manipulation the obstruction seemed to be removed, and such relief followed that I hoped the trouble was over, but I had to keep the patient on anodynes for a long while after getting a passage through the canal. Under mild purgative doses of mercury, the patient would improve for several days—seemingly convalescing well—when suddenly she would be seized with severe retching and vomiting, which could only be controlled by minute doses of calomel that arrested the vomiting after all other remedies would fail. After some time the patient passed some ten inches of the intestine, after which her improvement was constant, and she is now doing well—walking about, and gaining strength rapidly. The time of sloughing and discharge of the invaginated bowel was four weeks, when the whole invaginated portion passed off, at this time passing rather watery discharges. The patient lived on milk and other fluid diet during this critical period.

This case is interesting for its rarity, and as showing the peculiar efficacy of mercury in intestinal irritations. Pepsin and ingluvin were also used, with subnitrate of bismuth, alternating with tonics, stimulants and nutritious and bland diet to support the strength.

I have not mentioned certain complications, as of local peritonitis, etc., to which special treatment was directed. The part of the invaginated bowel which was discharged was evidently a portion of the small intestine.

